# Illinois State Dental Society

**Published:** 1887-07

**Authors:** E. E. Cady


### ILLINOIS STATE DENTAL SOCIETY.

Reported for the Independent Practitioner by E. E. Cady, D. D. S.

(Continued from page 309.)

### WEDNESDAY MORNING SESSION.

A telegram of congratulation and compliment was received from the President of the Northern Ohio Dental Association, and one of a similar nature sent in return.

In view of the distinguished services of Dr. G. V. Black to the entire profession, and to the society in particular, it was unanimously voted that his dues be remitted for life.

Dr. J. A. Swasey, Chairman of the Committee of Dental Art and Invention, mentioned in his report the following recent additions to the operating-room and laboratory: The Knapp blowpipe; gold lining for rubber-plates; the Shaw dental engine; Dunn's medical syringe; Woolley's root-canal dryer; Melotte's moldine and clamps; McKellop's dental register; Matteson's disk-guide; Ash & Sons' rubber-dam holder; Soloman's incandescent mouth-lamp; Taggart's automatic gas-regulator; Dr. Morrison's regulating appliance; Tibbit's bracket-table; Dr. Swasey's and Dr. Carpenter's rubber-dam clamps; Dr. Reed's and Dr. Bradley's matrices.

Dr. Homer Judd's paper, "Dead teeth in the jaws," was then read, the following being a summary: During the last few years various articles concerning this subject have appeared in the *Medical Record*. Some of these were from the pen of an eye and ear doctor named Sexton, and that of his assistant, Dr. Bartlett. These gentlemen report several cases of otalgia due to dental caries treated by them in the New York Eye and Ear Infirmary, in which report they say: "Among the poor, earache is in many cases due to simple otalgia arising from dental caries." This sage remark, in effect that earache is often due to a pain in the ear, could only have emanated from one who had received a thorough medical education. It is to be regretted, however, that the lesson thus attempted was not presented in a manner to impress those who saw it with the importance of understanding the intimate relation existing between the teeth and other organs. Much benefit might be derived from these cases had they been studied and reported by one who had some knowledge of medicine, and of what was required in a clinical report, to make it of value to others. Dental practitioners generally are enlightened upon this subject, and recognize the relationship here referred to as thoroughly established.

Dr. Sexton speaks of the "business" of dentistry, and the "work" of the dentist, and adds some senseless twaddle about the ills resulting from the use of artificial teeth. It must be understood, however, that the report here reviewed, and the articles from the pen of the editor of *The Record*, represent in their animus and general tone no phase of the intelligent public opinion of the medical profession.

A great majority of diseases of the teeth, mouth and jaws, have been studied as carefully and are treated by intelligent dentists as successfully as the diseases of any other location are treated by any other specialist, and there is little probability that material change in practice will be brought about, except as we ourselves acquire new facts by study and experiments. Eight cases are reported by the gentlemen referred to, but so little is said regarding treatment or subsequent progress, that we must conclude that the diagnosis was jumped at. The whole lesson taught by the eight cases reported amounts simply to this: People sometimes have earache, and sometimes those troubled with otalgia have also carious teeth in their jaws. In one solitary case reported, it seems probable the earache was dependent upon carious teeth. That otalgia is thus caused may be news to the readers of *The Record*, but those who have followed the dental literature of a few years past know that the teeth are frequently factors in diseases of the ear as well as of the eye and head, and that, on the other hand, toothache may be due to reflex action, having its cause in remote parts, especially the ovaries and uterus. Dr. Bartlett's report, therefore, proves nothing not already well known to the intelligent dentist, and his time would have been no more thrown away had he attempted to prove the value of quinine in intermittent fever. Dr. Sexton's choice of terms is rather primitive. We should expect him to speak of the surgeon's "tools," the chemist's "workshop," or the eye doctor's "kit," as he mentions the "business" of dentistry, and the dentist's "work." The same gentleman makes many ridiculous statements in discussing the results of the death of the pulp, saying, among other things: "Since the moment the pulp dies, thus cutting off the source of nutrition from the dentine, the part thus nourished must of necessity die also." And again: "It is true that through the periosteum alone the dentine may long derive some nourishment."

In the same number of *The Record*, the editor says: "Dentists who have a predilection for surgery would do well to bear in mind that a medical education and training are necessary accomplishments to acquire," etc., and by the same token it may be said that doctors who have a predilection for writing upon dental subjects should bear in mind that some knowledge of dentistry should be acquired before they attempt to teach it to others. The insinuation of the editor that the medical training of the dental student is insufficient, must be the result of ignorance on his part regarding our college curriculum. It is well known that in our leading colleges anatomy, physiology, pathology, chemistry, etc., are as thoroughly taught as in any medical college. Indeed, in the universities dental and medical students attend the same lectures on these subjects. This fact was recognized by the National Medical Association when it extended the hand of fellowship. Also by the International Medical Congress, which has given us honorable recognition as a specialty of medicine. Dr. Sexton speaks of the substitutes, or artificial teeth, as accomplishing much harm. He does not particularize any one material, but makes his declaration general. It is true that rubber often produces an irritation of the mucous membrane of the mouth, but this is no reason why all plate materials should be condemned, for the intelligent dentist knows that most objections can be overcome by using gold, platinum or aluminum. Bridge-work has not as yet received the endorsement of the more intelligent practitioners.

In answer to the question "Whether it is safe practice to retain dead teeth in the jaws" (Drs. Sexton, Shrady and Bartlett probably meaning teeth with dead pulps), an emphatic affirmative must be given. In mature life nature herself practically annihilates the pulp, and fills the canals with solid material without injury to general health. We daily see teeth from which the pulp has long been removed, which present all the appearances of healthy organs, and there is no evidence whatever going to show that even the dentine is deprived of vitality. We say, then, that intelligent dentistry does not advocate the retention of dead teeth in the jaws, and that pulpless teeth are not, necessarily, dead teeth. Abundant experience has demonstrated that a very great majority of pulpless teeth can be retained in the jaws, and made serviceable without danger to the general health.

DISCUSSION.

*Dr. Townsend*—I fully endorse all that the essayist has said, but the subject is scarcely one that calls for any discussion by this society, as experience and practice show that pulpless teeth can almost universally be retained in the jaws throughout life, if properly treated. There is no opportunity for debate in the matter. Alveolar abscess, and in fact all diseases of the teeth and mouth, are as successfully treated as the diseases of any other tissues of the body, and the principles underlying treatment are as fully understood by the dentist in his specialty, as by the physician in that which pertains to general medicine. It is true that artificial teeth, when the base is constructed of the cheaper materials, are in some instances injurious to health, but this can be obviated by having a plate of gold or continuous gum. Entirely to discard the use of artificial teeth would be as inconsequential as a refusal to wear flannel undergarments because some of the cheaper grades are colored with poisonous material.

*Dr. Marshall.*—I am sorry the essayist ridiculed the medical profession. Those who live in glass houses should not throw stones, and while it is true that physicians, like all other mortals, are liable to err, it is also true that dentists make as many mistakes in medicine as physicians do in dentistry. Let us drop this manner of referring to the mistakes of our medical brethren. Although nature partially fills the dentinal tubuli in some cases, it never does so completely.

*Dr. Ottofy.*—I do not agree with Dr. Marshall in the matter of ridicule. I think Dr. Judd has a perfect right to speak as he does, and hope it may stimulate the medical profession to a better knowledge of this subject. I believe the difference in amount of diseases produced by the various bases is very slight. It is simply a question of cleanliness. Foreign matter and microbes find a better lodging-place in the vegetable plates than they do in the mineral, and consequently the former require more persistent cleaning. It is essential that the gums and hard palate should be cleansed, as well as the plate.

*Dr. Sturgis.*—I approve of Dr. Judd's paper, and believe an essay of this kind will be beneficial to the medical profession. I am willing to discuss the matter with any physician, and am confident I shall be able to demonstrate as perfect knowledge of my profession as he can of his.

Dr. Koch and Dr. A.W. Freeman spoke to the same effect. Dr. Black explained the manner in which the discussion came up in *The Medical Record* two years ago. Editors of medical journals had not treated the matter fairly, and had not done justice to dentistry, for while the views of physicians were freely published, the papers written by dentists were suppressed. One of his sent to the *Medical Record* was thrown into the waste-basket, while a very unfavorable comment concerning it was published. It would have been better for all concerned had the discussion on both sides been given in full. We have ear troubles occurring from reflex nervous action, and hyperæmia of pulps of teeth, conditions which are well understood by the dental profession, but entirely ignored in this discussion by Dr. Sexton, *et al.*

*Dr. Sitherwood.*—If we lay ourselves open to ridicule, we should meet it unflinchingly. Perhaps such treatment is the best way to open our eyes, simply because it is unpleasant. It is true physicians make mistakes, but the fact that a single individual errs does not militate against the entire profession. They are our brethren, and we should cultivate the closest relationship with them.

Further discussion of Dr. Judd's paper was postponed until the afternoon session, and the society adjourned.

### WEDNESDAY AFTERNOON SESSION.

Dr. Black was called upon and said : I will first call your attention to the plants I made yesterday. You will remember that the broth in the tubes I then inoculated was clear like that I now hold in my hand, but the change caused is readily apparent to the naked eye, all of the plants made having grown. The first is the one taken from under the rubber plate, and is the species designated *Streptococcus.* As showing the reason why the tissues under these plates become sore when not kept clean, observe the acidity when tested with this litmus paper, the sterile broth you will remember being neutral, or nearly so. This acidity is caused by the physiological functions of the plant, the waste products being lactic acid. The acidity in these cases may continue until the growth stops. A rubber plate offers more rough places for the retention of these growths than does one of metal, and on this account alone do we have more sore mouths under the former.

My method of preparing the broth is as follows : To one-half pound of fresh, lean beef, add three pints of water, first having crushed the beef fine. Bring slowly to the boiling point in a water-bath, and boil for one hour. Filter off the fluid and sterilize it by boiling again in a water-bath. Now allow it to cool. Put in ʒ ij of pure pepsin and let it stand over night in a cool place. Boil again (in a water-bath) for one hour—it will be noticed in boiling this last time that there is considerable albuminous deposit, and the broth will have a white appearance. Filter again, neutralize with bicarbonate of soda, and if not clear filter again. Now place it in a glass flask, cork with cotton and put it in a steam-bath for one hour. The broth is then ready to place in tubes, provided it is clear after heating; if not, it must be filtered again. The tubes, thoroughly cleaned and stopped with cotton, are placed in an oven and the temperature gradually raised until the cotton is browned. The tubes being hot from the oven, and the broth taken direct from the steam-bath, the former are quickly filled and kept for four days at a temperature of 98 degrees Fahrenheit. At the end of that time, if clear and unclouded, they are ready for our purpose; but if any sign of growth is visible they must be discarded.

Gelatine : Add to 12 oz. of broth, as prepared, 60 grs. of well-dried agar-agar and 60 grs. of gelatine. Warm slowly, until the whole is dissolved, then bring to the boiling point in a water-bath. Filter and sterilize in the water-bath. Prepare and fill the tubes as described above. This gelatine ought to stand at 90 degrees. Place the tubes in a basket, and expose in a steam-chamber for half an hour. Repeat this the next day. After they are thoroughly cold, and the corks are dry, cover and tie with rubber-dam to prevent evaporation. Tubes prepared in this way will generally keep, but it is sometimes best to sterilize again after two days.

This gelatine is intended for surface planting, and the tubes should be so inclined as to give the largest surface possible. Most microbes grow better upon gelatine mixed with agar-agar than upon pure gelatine, but the colonies cannot be seen as well. For dry plate pure gelatine should be used.

The discussion of Dr. Judd's paper being continued, Dr. Noyes said: It appears to me that nearly enough time has been occupied in the discussion of medicine vs. dentistry, and we should now turn to the subject matter of the paper—retention of pulpless teeth in the jaws. It is important that the pulp cavity should receive proper attention in these cases, but the retention of pulpless teeth depends mainly upon the integrity and health of the peridental membrane. Success in implantation depends upon the same conditions.

Dr. Stevens said that he is not an advocate of Dr. Younger's process, and believes that in five years there will be very few successful cases in the mouth.

The report of Dr. M. L. Hanaford, of the Committee on Dental Science and Literature, was here read as bearing directly upon the subject. The following are the points of interest in the paper: The operators and the workers of to-day are far in advance of the theorists, and there are many matters of importance the theory of which is not fully understood or settled. Dr. Frank Abbott has stated that death of a pulp puts a stop to the absorption of the roots, which theory Dr. Sudduth denies, and asserts that absorption will continue, provided the peridental membrane be not in a purulent condition. Who is mistaken? This is a question of practical importance which science should settle.

Dr. Abbott recommends the use of gutta-percha dissolved in chloroform for filling root canals, and directs that a pellet of cotton wool be inserted in the apex of the root to prevent the filling material from being forced through the foramen. Another authority says this precaution is unnecessary, as a little gutta-percha driven through in this manner will do no harm. Who is right?

One authority directs the use of strongest carbolic acid in canals, while the Chicago Odontological Society claims that such treatment is not indicated. All this goes to prove that dentists are a class of hobby-riders. What we need is more careful investigation and study, and less dogmatism on the part of leaders and teachers.

Regarding the Herbst method, it was but natural that there should be much opposition in this country, but mature thought shows it to contain many good points.

It is unnecessary to discuss the method of Dr. Younger at present. While much about it seems improbable, we should remember that criticism is easy. Let us suspend our judgment and trust to time for the solution.

DISCUSSION.

*Dr. Black.*—A word regarding absorption. I have seen an extracted tooth, the roots of which had been filled with gold, where the roots themselves had been absorbed entirely away leaving the filling intact. I have seen the same phenomenon where the roots were filled with gutta percha. Sometimes absorption will take place midway of the roots, cutting them in two and leaving the apices remaining. This absorption always takes place from without, and does not depend upon the life of the root.

*Dr. Koch.*—Would an acrid condition of the pus cause absorption of a root?

*Dr. Black.*—Pus will not dissolve bone or absorb it.

*Dr. Koch.*—What becomes of the product of absorption?

*Dr. Black.*—It is in the blood.

*Dr. Marshall.*—Is there any difference in the process of the absorption of the permanent and temporary teeth?

*Dr. Black.*—I think not, though in the temporary teeth the process is more regular.

*Dr. Freeman.*—So you regard the 5 per cent. solution of carbolic acid strong enough for use in these cases?

*Dr. Black.*—I should prefer it stronger.

*Dr. Freeman.*—Do you like the solution of bichloride of mercury better?

*Dr. Black.*—It destroys steel, and hence is not so good as carbolic acid, especially when broaches are used.

*Dr. Harlan.*—We are losing sight of the subject at issue. There are many methods of treating pulpless teeth. One that I take exception to is presented in Dr. Hanaford's paper, and refers to the use of carbolic acid, full strength, in root canals. Carbolic acid, in full strength, is escharotic and antiseptic. What need have we of an antiseptic in a tooth after it is brought into a condition to fill? We require nothing but complete dryness and perfect filling of the root from apex to pulp-chamber. The question of filling the apex is a simple one. We must have a material that is completely impervious to moisture. I do not object to the use of paraffine, shellac, gold, gutta percha, etc., but I do object decidedly to the use of cotton or any other absorbent substance.

*Dr. Marriner*—Cited a case of absorption similar to those mentioned by Dr. Black.

*Dr. Judd.*—It is generally believed that Dr. Younger was the first to implant teeth, but similar experiments were performed by a man named Litzskerlk, in Germany, about fifteen years ago. Like all Germans, he did his work thoroughly. He found teeth thus implanted would become firm, but that in time holes appeared in the roots, and a projection into these from the alveolar walls retained the teeth. According to the gentleman's operation there was no vital connection.

EVENING SESSION.

Dr. Koch, Chairman of Committee on Dental Science and Literature, here made his report. He said that the establishment of the new dental journals of the past year was uncalled for. The profession needs not more journals, but better ones, with more carefully condensed matter.

The practice of conferring degrees by State boards should be abolished. The advance in comprehension of the theory of decay and the inward knowledge of the character of the peridental membrane was a matter of congratulation for the profession. Implantation appears to be the only true scientific advance of the year, but even this will require the test of time to determine its value. All the members were urged to study and experiment in that special line for which they felt most inclination and aptitude.

Dr. L. C. Ingersoll then read his paper on "Medicinal Stimulants." He commenced by saying that this is a laggard world. Mankind must have stimulants. The animal creation, when domesticated, must be stimulated. Even the soil needs stimulation. The entire world craves stimulants, in proof of which witness the demand for alcohol, tobacco, tea and coffee. On every dinner table is the castor with its variety of stimulating condiments—mustard, cayenne pepper, black-pepper, horse-radish and pepper-sauce.

Human nature manifests three sorts of functions—mental, moral and physical—each demanding its appropriate stimuli. No function works uniformly up to its highest standard of normality, hence the demand for an appropriate stimulus, either natural or artificial. A stimulant is a medicine which has the effect to goad on the functions to more complete service. Retardation of the blood is known to be one of the earliest histological manifestations of inflammation. A stimulant which will overcome the retardation and quicken the blood into normal flow will arrest the inflammatory tendencies and bring about resolution.

The most common indications of disease which the dentist is called upon to treat are pain and swelling. The swelling, though not perceptible to the eye, is known to the patient by a sense of fullness and pressure. This is caused by an over-fullness of the blood-vessels, which in turn is caused by retardation of the blood-current. This condition plainly calls for stimulants, with promise of success in reducing the swelling and attendant symptoms. The sense of pain is caused by unusual pressure of the over-full blood vessels upon the filaments of nerves that supply the parts; hence the stimulant that will quicken the blood-flow will relieve the pain, and for this reason one of the essential properties of a pain obtundent, where the conditions are as above stated, is stimulation.

In this connection we must realize that the stimulating effect is produced by irritation. Stimulants must be sensibly felt. They must smart, burn and irritate the surface to which they are applied. The soothing sensation and the relief from pain is a secondary effect.

This brings us to that modification of the effect of stimulants by which anæsthesia is produced. Alcohol is a powerful stimulant, and is also sedative and anæsthetic. Its primary effect is stimulant, its secondary anæsthetic. Under the former men got boisterously drunk; under the last, dead drunk. This latter is the secondary effect which follows stimulation. Almost the same action is found in using ether and chloroform. In this connection we observe the opposite effects produced by the same drug, depending upon the amount administered, the small quantity acting as a stimulant, the larger doses, according to size, acting as a sedative, narcotic or anæsthetic.

We also rely upon stimulants for a tonic effect. Tonics insensibly promote healthy action and bodily vigor, and differ from stimulants, not in kind but in degree, the latter producing a sensible impression, while the impression of tonics is insensible. A medicine may, therefore, be both a stimulant and a tonic. As a stimulant it is mild and without any irritating effect, and this is what constitutes it a tonic. We say of a pure atmosphere that it is both stimulating and tonic. Then by dilution any stimulating agent may be reduced to a tonic.

It has of late years come to be the general opinion that little else than antiseptics disinfectants and germicides are required in healing diseases of whatever nature. For this reason it is not strange that the importance of other medicinal agents should be overlooked. If what I have already stated be true, however, something more is needed than antiseptic treatment of disease.

The enthusiasm attending the late developments of science has demanded a revision of our materia medica, that throws out many old and well-tried remedies which have proved reliable for years, because they will not stand the test as germicides. Their value is not judged by the results of actual practice, but is based on experimentation in the laboratory, where natural causes and vital reactions are forced to give way to artificial causes and chemical reactions. In these tests vital functions have been ignored. As a result of these artificial methods, preference has been given a certain class of medicines solely because they are non-irritants. Such a medicine as iodoform, containing a condensation of disgusting odors such as sulphuretted hydrogen, were inflicted upon human olfactories and are sufficient to drive patient and operator forever asunder after the first treatment with the insulting medicine, requiring also that it be kept on a platform outside the office, where a free circulation of air between it and the walls of the building may protect the apartments within from being saturated with the foul, profanity-provoking odor. This medicine makes special claims for preference over carbolic acid, creosote and phenol sodique, because it is a non-irritant. Failing in this, it was changed to iodol, an odorless non-irritant robbed of seventy-five per cent. of iodine. Bichloride of mercury and hydro-naphthol are brought into special favor, not alone on their merits as disinfectants and germicides, but because non-irritants, which is only another word for non-stimulant. Stimulation and irritation serve such an important purpose in arousing functional action and equalizing the circulation, that I look with little favor on non-irritant antiseptics, except in cases where stimulant disinfectants have proved unsuccessful. In certain cases in the sick room, and for general hygienic purposes, it is of no importance that the antiseptic be stimulant. For sewers and cesspools only the antiseptic and disinfectant properties are required. But the same medicines are not proper for general use in dental practice.

We often find septic processes to be overcome in contact with and in the midst of the living tissues. We desire here to produce, not merely an aseptic condition, but an antiseptic condition, which is accomplished by stimulating the functions of the tissues involved. This is true in cases of ulceration. If it were proved beyond doubt that all disease has its origin in micro-organisms, and should a germicide be discovered for every species of microbes that are poisonous to the human body, it would still remain true that disinfectants and germicides are not, unaided, sufficient to cure sporadic disease, for the functions are deranged and must be restored to their normal action. In some cases it is true, however, that removal of the cause will effect a cure; but this is not always sufficient, for if it be a case in which the cause has long been operative the functions have adjusted themselves to the abnormal condition of the tissues until a sort of functional momentum is acquired, and they run on continuously after the cause is removed. This is what we term "second nature"—the formation of a functional habitude by the continuance of abnormal conditions. The only mode of restoration, therefore, is to change the functions, though to accomplish it we must destroy the tissue itself. But it may be accomplished in many cases short of this by a powerful stimulus.

What is here stated regarding alcoholic stimulants is said from a medicinal standpoint only. The plea for stimulants as medicine is a plea in behalf of the sick and diseased. The well have no right to demand them.

DISCUSSION.

*Dr. Newkirk.*—I consider the paper an excellent one, but would leave the discussion of that part relating to antiseptics and disinfectants to those better qualified to do it justice—to Dr. Black for instance. The proper place for stimulants in inflammatory stages is not well understood. Disease is aggravated instead of cured, unless the remedy is applied at the proper time. Alcohol is a stimulant only when administered in small quantities. The effect of alcohol, as shown in the capillaries, nerves and brain, is not stimulant.

*Dr. Black.*—The subject of antiseptics is one I hardly feel like talking about as yet. We have not got a fair hold of it as yet. We have plenty of good remedies, however, particularly those of a local nature. I think it hardly necessary that these should be stimulant. We really know little how poisons affect, and antiseptics must be poisonous. We may combat micro-organisms in other than antiseptic ways. For instance, a man with boils is given alkalies. Pus from these boils, if made alkaline, will not grow. We may by proper tonics produce a condition of the system that will banish micro-organism, and to this we must turn our attention.

Next in order was the paper by Dr. A. W. Harlan on "Practical Therapeutics, with Notes on the Application of Special Drugs."

The blunders of the empiric, and the subsequent study of physiological action, may be called practical scientific therapeutics. The workers in this field are increasing, as may be noticed by reference to the labors of the dozen or more in this country and abroad who have written on the subject. It is less than two hundred years since the dawn of physiological therapeutics, and we are indebted to Albrecht Von Haller for the following: "In the first place, the remedy is to be tried on the healthy body without any foreign substance mixed with it. Having been examined as to its order and taste, a small dose is to be taken and the attention directed to all effects which thereafter occur: such as upon the pulse, the temperature, the respiration and the excretions. Having thereby adduced their obvious phenomena in health, you may pass on to experiment upon the sick body."

The thralldom of the past ages of ignorance, of secret powders, essences and elixirs has been shaken off, and instead we have the open doors of experimental laboratories, and are assisted in our search for knowledge, instead of being enveloped in a dense cloud of ignorant charlatanism. In this age of enlightenment it is the duty of every dentist to know for himself why he uses a drug or chemical, and not allow himself to be misled by the pretentious claims of those who have a mercenary object in selling them. I

feel that only by iteration and reiteration can the basal principles of treatment be thoroughly inculcated, and if I can assist in the great work which our leader, Dr. Black, is engaged in—if he can discuss the cause of disease I will supply the remedy to prevent or cure it—and therein will be found my compensation. As our knowledge of pathology increases we must enlarge our understanding of therapeutics and materia medica.

Iodoform is valuable in surgery, but its odor is disagreeable; it is also toxic and irritant under certain conditions, therefore salol and iodol have been recommended as substitutes, both being agreeable and without odor. Salol is obtained by the action of carbolic acid on salicylic acid, and is soluble in alcohol, though not in water. The crystals applied to the cavity of a living tooth causes no pain. Salol is tasteless, odorless and non-irritant. Its antiseptic and disinfectant properties are not impaired by its lack of solubility in water. It is indicated in acute and chronic gingivitis, and will destroy all kinds of microbes in dilute solutions. It will prevent sepsis after operations, when dusted over the surface of wounds. It is useful in acute rheumatism, in catarrh of the bladder, and for the disinfection of the intestinal canal in typhoid fever. It may be administered in doses of from gr. x to ℥ ij.

Iodol is an expensive drug, its cost being at this time almost eight times that of iodoform. It is indicated wherever iodoform was formerly used, but is more bulky. It is particularly useful in sluggish abscesses and in gangrenous conditions of the mucous membrane and gums.

Ozonic ether, a thirty-volume solution of peroxide of hydrogen in ether, to which five per cent. of alcohol is added, is another useful combination. It is freely soluble in water, and much more stable than the ordinary $H_2 O_2$. It may be used for the same purposes locally, except on the conjunctiva and the urethral mucous membrane. For all purulent discharges it is useful as a local astringent. It is colorless, odorless, painless, and is not poisonous.

Lanolin is easily absorbed by the unbroken skin, and is a convenient vehicle for the incorporation of substances for the relief of pain when applied externally. In persistent neuralgia the extract of gelseminum or aconitum may be thus used. Aconite is highly esteemed as a liniment, and even aconitea has been recommended, but for general local use it is too powerful to be used by the inexperienced. It would be safe, however, to combine $\frac{1}{4}$ gr. of aconitea with ten minims of rectified spirit, and add to it one ounce of lanolin. Five or ten grains of this ointment might then be rubbed on the affected part, care being taken to protect with a glove the fingers used for applying the remedy. Lanolin does not become rancid, and will take up more than an equal volume of water. Cocoaine and many other salts may be rubbed up in it.

Menthol is too little used as a local pain obtunder. The following substances liquefy when rubbed together: Equal parts of menthol and thymol crystals; equal parts of menthol and absolute phenol; equal parts of menthol and chloral hydrate; three parts of menthol and two of gum camphor; two parts of menthol and one of croton-chloral-hydrate; two parts of menthol and one each of carbolic acid and croton-chloral-hydrate. These form colorless, oily fluids, agreeable to patient and operator. They will quickly relieve the pain of an aching pulp, and are useful as local anæsthetics, not producing an eschar.

I will close by saying: "Physiological experimentation with drugs must be the basis of their therapeutical employment."

DISCUSSION.

*Dr. Sitherwood.*—It is my practice to use medicines from which the most benefit may be derived from the smallest quantity. I have nearly abandoned the use of creosote, carbolic acid, iodoform, and several others, on account of their disagreeable odors, etc. I like the capiscum plasters for their stimulating effect.

I use eucalyptus and aconite in glycerine. Glycerine is an excellent article used in this manner. I use eugenol exclusively, where I formerly applied oil of cloves, or remedies of a similar nature. It is especially agreeable in the mouths of children, as a dressing for exposed pulps. Phenol sodique and glycerine, equal parts, is better than the former alone. I use a ligature instead of a knife in excising hypertrophied gums and removing small, soft tumors. In pyorrhœa alveolaris the following is excellent:

Glycerine ℥ j.

Eucalyptol gt. x.

Adjourned.

(TO BE CONTINUED).

